# The Association of Heterozygous p.R4810K of RNF213 and Long-Term Unfavorable Outcomes after Encephaloduroarteriosynangiosis in Chinese Pediatric Patients with Moyamoya Disease

**DOI:** 10.1155/2024/1844190

**Published:** 2024-04-24

**Authors:** Qingbao Guo, Fangbin Hao, Qian-Nan Wang, Jingjie Li, Shitong Liu, Zhengxing Zou, Simeng Liu, Xiaopeng Wang, Dan Yu, Gan Gao, Qian Zhang, Songtao Pei, Jie Feng, Rimiao Yang, Minjie Wang, Heguan Fu, Cong Han, Xiangyang Bao, Lian Duan

**Affiliations:** ^1^Department of Neurosurgery, The Fifth Medical Centre, Chinese PLA General Hospital, Beijing, China; ^2^Medical School of Chinese PLA, Beijing, China; ^3^Department of Neurosurgery, Chinese PLA General Hospital, Beijing, China; ^4^Department of Neurosurgery, The Eighth Medical Centre, Chinese PLA General Hospital, Beijing, China

## Abstract

**Background:**

Previous studies have established that heterozygous mutation for the p.R4810K variant can influence the severity of the clinical phenotype in patients with moyamoya disease (MMD) at disease onset. However, the relationship between the p.R4810K variant and the clinical phenotype of long-term unfavorable outcomes in Chinese pediatric patients remains unclear.

**Objectives:**

The primary aim of this study was to examine the association of heterozygous p.R4810K of RNF213 and long-term unfavorable outcomes after encephaloduroarteriosynangiosis (EDAS) in Chinese pediatric patients with MMD.

**Method:**

In this retrospective cohort study, we included 259 pediatric patients with MMD who possessed the known p.R4810K genotype. These individuals underwent EDAS along with genotyping analysis for p.R4810K via a TaqMan probe and the QuantStudio 6 Flex Real-Time PCR System. Subsequently, we evaluated their long-term outcomes. The variables we assessed were age at diagnosis, gender, p.R4810K genotypes, initial modified Rankin scale (mRS), clinical manifestations (such as hemorrhage and ischemia), posterior cerebral artery (PCA) involvement combined with angiographic stage, and their history of risk factors like hyperlipidemia and hyperhomocysteinemia. Furthermore, we scrutinized long-term unfavorable outcomes using both univariate analyses and multivariate logistic regression to identify independent predictive factors.

**Results:**

This study enrolled 259 Chinese pediatric patients with MMD, which included both newly and previously diagnosed cases, who underwent EDAS. The cohort comprised 130 male participants (50.19%) and 129 female participants (49.81%), with a median onset age of 8 years (median, IQR: 6-12 years). Among these patients, homozygous mutations were exceptionally rare, identified in only 4 individuals (1.54%), while the prevalence of heterozygous mutations was relatively higher, observed in 85 children (32.82%). The multivariate logistic regression showed that several factors were significantly associated with long-term unfavorable outcomes: older age at diagnosis (OR, 0.82 [95% CI, 0.7-0.96], *P* = 0.014), onset with hematoma (OR, 12.76 [95% CI, 1.52-106.89], *P* = 0.019), initial mRS (OR, 24.53 [95% CI, 6.51-92.41], *P* < 0.001), perioperative infarction (OR, 22.16 [95% CI, 1.45-337.96], *P* = 0.026), and infarction during follow-up (OR, 14.5 [95% CI, 2.04-103.12], *P* = 0.008). Furthermore, the cumulative incidence of initial infarction suggested that pediatric patients with homozygous or heterozygous mutations typically present at a younger age and exhibit a higher incidence of initial infarction compared to those carrying wild-type genotypes.

**Conclusions:**

The study suggests that the p.R4810K variant is associated with the onset age of MMD in Chinese pediatric patients, potentially impacting long-term outcomes. Surprisingly low recurrent stroke rates were observed across all genotypes, including homozygous individuals for the pathogenic variant, indicating that nongenetic factors may also play a role in the course and outcomes of MMD in this population.

## 1. Introduction

Moyamoya disease (MMD) is a chronic cerebrovascular disorder characterized by the progressive stenosis of the terminal segment of the internal carotid artery, as well as the proximal anterior and middle cerebral arteries, accompanied by the development of compensatory collateral vessels at the brain's base [1]. This condition can lead to severe neurological deficits and cognitive impairments if untreated [2]. Clinical manifestations in MMD vary with age [3]. Adults often experience intracranial hemorrhages (∼46%) [4], whereas pediatric patients typically present with cerebral ischemic symptoms, such as transient ischemic attacks and cerebral infarctions. Moreover, pediatric patients after a transient ischemic attack (TIA) are at a higher risk of sustaining complete infarcts that can result in brain developmental injury and adverse clinical outcomes compared to adults [5]. Encephaloduroarteriosynangiosis (EDAS) has been shown to yield favorable outcomes in pediatric MMD patients by reducing ischemic events [3, 6]. Consequently, early diagnosis and timely surgical intervention are crucial for improving pediatric neurologic prognoses.

The etiology of MMD has historically been challenging to pinpoint, chiefly because diagnosis necessitates the exclusion of other potential medical causes. However, a pivotal discovery in 2011 identified the RNF213 gene's p.R4810K locus, known as mysterin, as a genetic susceptibility factor for MMD. This correlation between RNF213 and MMD has shed considerable light on the genetic contributors to this disorder. Subsequent studies have shown that RNF213 codes for a significant E3 ubiquitin ligase, elucidating the molecular pathways implicated in MMD [7].

Genetic studies reveal that approximately 95% of Japanese familial cases [8], 80% of Japanese and Korean sporadic cases, [8, 9], and 20% of Chinese sporadic cases harbor the variant [10]. The clinical significance of the p.R4810K variant is evident, as individuals with the AA genotype usually have an early onset of MMD and more pronounced symptoms. These studies have highlighted the importance of this variant in understanding the progression and severity of MMD [2]. Prognostic factors for pediatric MMD include preoperative cerebral infarctions, early onset, intellectual impairment, and progressing posterior cerebral artery (PCA) stenosis [11, 12]. However, it is currently unclear whether the p.R4810K variant has an impact on long-term unfavorable outcomes in pediatric patients with MMD following EDAS. The study hypothesizes that the presence of the p.R4810K variant in Chinese pediatric patients with MMD may be associated with long-term unfavorable outcomes following EDAS. It is crucial to explore this relationship further to personalize treatment strategies and prognostic assessments, particularly in the Chinese pediatric population, where the prevalence and clinical behavior of MMD may differ from other ethnic groups. This study is aimed at investigating its association with long-term unfavorable outcomes after EDAS in Chinese pediatric patients with MMD. By elucidating the genetic influence on surgical success and patient prognosis, healthcare providers may be better equipped to manage this complex and debilitating disease.

## 2. Methods

### 2.1. Sample Size

Considering that the incidence rate of adverse outcomes in children with MMD is known to be 0.5% [11], with the permissible error (*δ*) for this survey being set at 0.01 and a type I error (*α*) for the hypothesis test at 0.05, the sample size calculation formula yields *n* = (*z*1 − *α*/*δ*)^2^*p*(1 − *p*), where *z*1 − *α*/2 = 1.96. This results in a calculated sample size of 192. Assuming an attrition rate of 20%, a minimum sample size of 240 is required. Our cohort consists of 259 Chinese children with MMD.

### 2.2. Study Design and Patients

This study is a single-center, retrospective, cross-sectional, and longitudinal analysis. It included 259 Chinese pediatric patients under 18 years of age, either newly or previously diagnosed with MMD, who underwent EDAS. These patients were consecutively recruited from the Fifth Medical Center of the Chinese People's Liberation Army (PLA) General Hospital between January 2012 and December 2018.

This study was approved by the Ethics Committee of the Fifth Medical Center of the PLA General Hospital (approval number: KY-2020-9-22) and adhered to the Declaration of Helsinki. Due to the retrospective of the data, an exemption from obtaining informed consent was granted. This study was reported in line with the STROCSS criteria. The inclusion criteria were as follows: (1) MMD diagnosed using digital subtraction angiography (DSA) or magnetic resonance angiography (MRA) in accordance with the Japanese guidelines for the management of MMD [13], (2) age < 18 years, (3) prior EDAS treatment, (4) a follow-up period of longer than 5 years, and (5) a genotyping analysis for the p.R4810K variant conducted. The exclusion criteria were as follows: (1) incomplete basic information, (2) absence of prior surgical intervention, (3) age ≥ 18 years, (4) presentation with moyamoya syndrome (MMS), and (5) the presence of central nervous system tumors, severe brain injury, prior craniotomy, or related conditions.

For patients who underwent bilateral EDAS on both hemispheres, only data from the hemisphere that underwent the initial surgery were included for analysis in this study. The Institutional Review Board and Ethics Committee of the Fifth Medical Center of the Chinese PLA General Hospital granted approval for this study, and appropriate informed consent was obtained from either the patients or their guardians.

In this study, we gathered data on various clinical variables that could potentially predict unfavorable outcomes. These variables include age at diagnosis, gender, p.R4810K genotypes, initial mRS, clinical manifestations (such as hemorrhage and ischemia), PCA involvement combined with angiographic stage, and their history of risk factors like hyperlipidemia and hyperhomocysteinemia.

### 2.3. Surgical Implementation

The EDAS procedure was carried out consistently by the same surgical team. A critical procedure involves harvesting the scalp artery along with the galeated band, ensuring the preservation of the distal and proximal arteries. These harvested arteries are then [14] grafted into a narrow linear dural opening created through an osteotomy. The technique used in this process was first described by Matsushima et al. [15].

### 2.4. Cross-Sectional and Longitudinal Analysis of Clinical Outcomes and Stroke Event Probabilities

#### 2.4.1. Primary Outcome

The primary outcome of the study is the functional status of patients as evaluated by the mRS. This assessment can help determine the effectiveness of treatment and predict the prognosis for patients. The scale ranges from 0 to 6, with higher scores indicating more severe functional impairment. Specifically, the primary outcome is the proportion of patients with a mRS score of ≥2, indicating moderate disability.

#### 2.4.2. Secondary Outcome

The secondary outcome of the study is the occurrence of new stroke events, which include both ischemic and hemorrhagic strokes, that manifest with neurological symptoms more than 30 days after the EDAS procedure. The definition of a new stroke event in this study remained consistent with previous large-scale studies [14].

These outcomes are crucial in assessing the efficacy of the intervention, predicting patient prognosis, and guiding future treatment strategies.

### 2.5. DNA Extraction and Single-Nucleotide Polymorphism Genotyping

Following obtaining informed consent, 10 mL of peripheral vein blood was collected from pediatric patients with MMD. The blood samples were then preserved in EDTA Na_4_ anticoagulant tubes and subsequently frozen at -80°C until further analysis. Subsequently, genomic DNA was extracted from these samples using the Blood Genetic DNA Mini Kit (CWBIO, Beijing, China). The DNA concentration (4303 DNA) was determined using the NanoDrop 2000 (Thermo Fisher Scientific, Waltham, MA). The DNA was then diluted to create working solutions with a concentration of 5 ng/*μ*L for subsequent genotyping and validation purposes.

All participants underwent genotyping analysis for p.R4810K using a TaqMan probe (TaqMan SNP Genotyping Assays, Applied Biosystems in Foster City, CA) and a QuantStudio 6 Flex Real-Time PCR System (Applied Biosystems). The comprehensive system consisted of 5 *μ*L : 2.0 *μ*L of purified genomic DNA, 2.5 *μ*L of TaqPath ProAmp Master Mixes (Applied Biosystems), 0.1 *μ*L of 40x SNP genotyping assay, and 0.4 *μ*L of deoxyribonuclease-free water. The appropriate PCR thermal cycling conditions were earmarked as a standby period of 5 minutes for initial enzyme activation/denaturing, 40 sequences of 5 seconds at 95°C for denaturation, and 60 seconds at 60°C for annealing and extension. At the end of each PCR amplification, a final plate read was carried out using the QuantStudio 6 Flex Real-Time PCR System. The genotype of every sample was validated based on the observed fluorescence signals.

### 2.6. Statistical Analysis

A total of 259 pediatric patients were divided into three groups based on their p.R4810K genotype: homozygous p.R4810K (AA) group (*n* = 4), heterozygous p.R4810K (GA) group (*n* = 85), and wild-type p.R4810K (GG) group (*n* = 170). Due to the low occurrence of the AA genotype, the subsequent logistic regression analysis only focused on the GA and GG groups. The study investigated the clinical characteristics and radiological evaluations of each group. Categorical variables were presented as counts and percentages, while continuous variables were presented as mean and standard deviation. We used chi-square tests or Fisher's exact tests for categorical variables to determine if there were significant differences in proportion between groups. For continuous variables, we employed independent samples *t*-tests or Mann–Whitney *U* tests for nonparametric distributions to compare the means between two independent groups. When assessing normality, we adopted both graphical and statistical methods to provide a comprehensive evaluation, including Q-Q (quantile-quantile) plots and the Shapiro-Wilk test. A logistic regression model was built to predict long-term unfavorable outcomes based on the baseline clinical and imaging variables. In the multivariate logistic regression model, a significance level of *P* < 0.05 was considered statistically significant. The statistical analysis was performed using the R programming language and environment (R Core Team, 2022). The R Foundation for Statistical Computing in Vienna, Austria, provided the necessary tools (URL: https://www.R-project.org/). Additionally, the J MedCalc® Statistical Software version 20.218 (MedCalc Software Ltd, Ostend, Belgium; https://www.medcalc.org; 2023) was utilized for analysis.

The definitions of the evaluated items in this study have been established. The onset of symptoms was determined based on the clinical symptoms observed during the first attack or the presence of infarction as indicated by MRI results. In cases of epilepsy and TIA in pediatric patients, if a subsequent MRI study confirmed the presence of infarction, it was categorized as such. In cases where TIA progressed to epilepsy, it was defined as epilepsy. Intracranial hemorrhage was defined as the detection of any symptoms on the initial computed tomography scan after the occurrence of intracranial hemorrhage. It was considered an intracranial hemorrhage even if there were other symptoms prior to the bleeding event.

The duration between the diagnosis of MMD and the surgery was defined as the time elapsed from the diagnosis to the surgical intervention.

## 3. Results

### 3.1. Patient Characteristics

**Figure 1:**
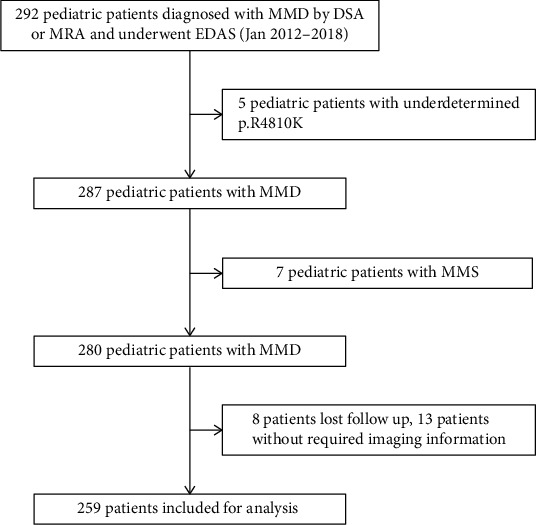
Flowchart of patient inclusion.

From Jan 2012 to 2018, a total of 292 pediatric individuals were diagnosed with MMD based on DSA or MRA. These patients also underwent EDAS and had their p.R4810K genotype identified. However, the p.R4810K genotype could not be determined for 5 of these patients, accounting for 1.71% of the total cohort. A total of 28 patients were excluded from the study. Thirteen of them had incomplete or poor-quality image information, while 8 were lost to follow-up. Additionally, 7 patients diagnosed with MMS were excluded from the study. Therefore, a total of 259 patients, corresponding to 259 hemispheres, were included in the study (Figure 1). Following long-term follow-up, 225 patients (86.87%) achieved favorable outcomes, while 34 patients (13.13%) experienced unfavorable outcomes. The baseline characteristics of these patients and hemispheres are presented in Table 1. The cohort comprised 130 male participants (50.19%) and 129 female participants (49.81%), with a median onset age of 8 years (median, IQR: 6-12 years). Throughout the long-term follow-up period, nine hemispheres (3.47%) experienced cerebrovascular events again, including 1 hemisphere (0.39%) with hemorrhaging and 8 hemispheres (3.09%) with infarction. There were no significant differences in baseline characteristics between the unfavorable and favorable outcome groups (*P* > 0.05; Table 2). These characteristics included gender, onset with infarction, onset→operation, p.R4810K genotypes, hyperlipidemia, hyperhomocysteinemia, family history, laterality, PCA unilateral involvement, PCA bilateral involvement, and Suzuki stage. However, there were significant differences in age at onset, onset with hematoma, waiting infarction, Initial mRS, and perioperative infarction (*P* < 0.05; Table 2). Since these variables were already included in the model during the analysis, further adjustments were not necessary.

### 3.2. Univariate Logistic Regression Analysis

Univariate logistic regression analysis was conducted to identify the association between pediatric clinical variables and long-term unfavorable outcomes. The results indicated that age at onset (OR, 0.87 [95% CI, 0.79-0.97], *P* = 0.009), onset with hematoma (OR, 17.44 [95% CI, 4.9-62.07], *P* < 0.001), onset→operation (ref: <2 mons) (OR, 0.44 [95% CI, 0.2-0.97], *P* = 0.042), waiting infarction (OR, 4.94 [95% CI, 2.26-10.77], *P* < 0.001), initial mRS (ref: <2) (OR, 38.36 [95% CI, 14.93-98.61], *P* < 0.001), and perioperative infarction (OR, 22.1 [95% CI, 2.23-219.35], *P* = 0.008) were identified as risk factors for the long-term unfavorable outcomes (Table 3).

### 3.3. Multivariate Logistic Regression Analysis

In the multivariate logistic regression analysis, we incorporated variables with *P* < 0.1 from the univariate analysis. This analysis revealed that older age at diagnosis (OR, 0.82 [95% CI, 0.7-0.96], *P* = 0.014), onset with hematoma (OR, 12.76 [95% CI, 1.52-106.89], *P* = 0.019), initial mRS (OR, 24.53 [95% CI, 6.51-92.41], *P* < 0.001), perioperative infarction (OR, 22.16 [95% CI, 1.45-337.96], *P* = 0.026), and infarction during follow-up (OR, 14.5 [95% CI, 2.04-103.12], *P* = 0.008) were significant predictive factors for the long-term unfavorable outcomes (Table 4).

### 3.4. Longitudinal Analysis

During the follow-up period, 8 infarctions occurred (Table 5) in the operated hemispheres, of which 2 were silent infarctions without neurological symptoms and 1 hematoma occurred in the operated hemispheres. The median time of infarction during follow-up was 4.95 (3.96, 7.19) years. The operated brain hemispheres showed an annual risk of 0.30% of symptomatic infarction and 0.05% of hemorrhage.

### 3.5. Carrying Rate of RNF213 p.R4810K in Pediatric Patients

In Chinese pediatric patients with MMD, the prevalence of homozygous AA mutations is extremely low, as observed in only 4 (1.54%) out of 259 pediatric patients. On the other hand, the carrier rate of GA heterozygous mutations is relatively high, with 85 (32.82%) out of 259 children exhibiting this genotype (Table 1).

### 3.6. Correlation between the Clinical Phenotype of Long-Term Unfavorable Outcomes and RNF213 p.R4810K Genotype

**Figure 2:**
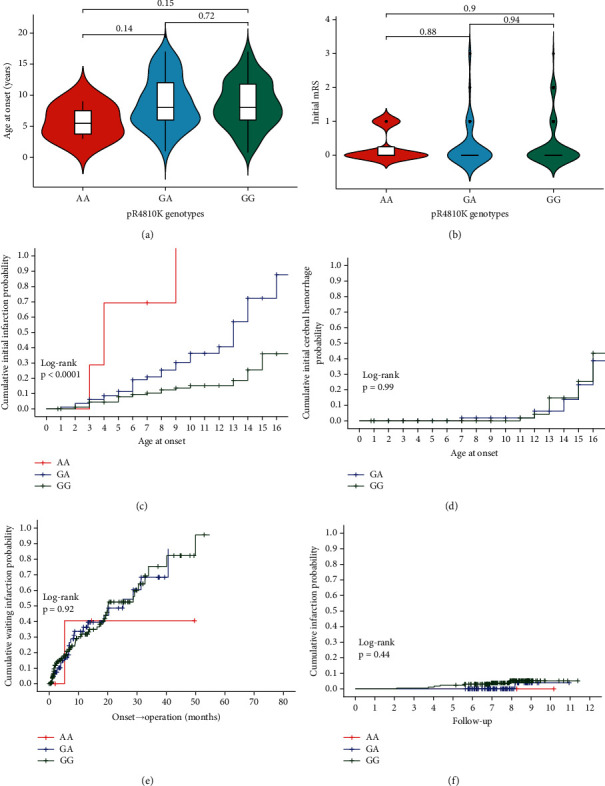
Correlation between the clinical phenotype of long-term unfavorable outcomes among three pediatric patient groups with different genotypes (AA, GA, and GG) and RNF213 p.R4810K genotype. (a) The violin chart plot of age at onset for three pediatric patient groups with different genotypes (AA, GA, and GG) of the p.R4810K variant in moyamoya disease. (b) The violin chart plot of initial mRS for three pediatric patient groups with different genotypes (AA, GA, and GG) of the p.R4810K variant in moyamoya disease. (c) The cumulative initial infarction incidence curve of MMD in three patient groups with different genotypes (AA, GA, and GG) of the p.R4810K variant. (d) The cumulative initial hematoma incidence curve of MMD in three patient groups with different genotypes (AA, GA, and GG) of the p.R4810K variant. (e) The cumulative waiting infarction incidence curve of MMD in three patient groups with different genotypes (AA, GA, and GG) of the p.R4810K variant. (f) The cumulative infarction during follow-up incidence curve of MMD in three patient groups with different genotypes (AA, GA, and GG) of the p.R4810K variant.

We analyzed the clinical characteristics of long-term unfavorable outcomes in pediatric patients with MMD based on their p.R4810K genotype. The patients were classified into three groups: the wild type (GG), heterozygotes (GA), and homozygotes (AA). The median age of onset for MMD varied according to genotype: 5.5 years for AA, 8 years for GA, and 8 years for GG. Although there appears to be an increasing trend in the age of onset from AA to GA and GG, the difference is not statistically significant (Figure 2(a)). Furthermore, there was no significant difference in the initial mRS scores among the three genotype groups (Figure 2(b)). The cumulative incidence of initial infarction in individuals with MMD was generally higher in those with AA and GA genotypes compared to the GG genotype (*P* < 0.05, Figure 2(c)). However, there was no significant difference in cumulative incidence of the initial hematoma between individuals with GA and GG genotypes (*P* = 0.99, Figure 2(d)). Moreover, there were no significant differences observed in the cumulative incidence of waiting infarction among the three genotype groups (*P* = 0.92, Figure 2(e)) or in the occurrence of infarction during follow-up (*P* = 0.44, Figure 2(f)).

## 4. Discussion

In this study, we present a comprehensive analysis of the p.R4810K variant in RNF213 and its association with long-term unfavorable outcomes following EDAS in Chinese pediatric patients with MMD. Considering the rarity of AA among Chinese children, we concentrated our logistic regression analysis on the comparative impact of GA and GG on long-term prognosis. The study documented a low incidence of recurrent strokes post-EDAS during a median follow-up of 7.73 years (range 6.88 to 8.52 years), which was consistent across all p.R4810K genotypes. Remarkably, even in homozygous variants, anticipated to exert the most substantial genetic influence, we detected no significant differences in the occurrence of recurrent strokes or final mRS scores compared with other genotypes. The findings indicate that the key predictors of long-term adverse outcomes include age at diagnosis, onset with hematoma, initial mRS, perioperative infarction, and infarction during follow-up. Our findings underscore the necessity of early surgical intervention. Independent of genetic variants, prompt surgery, meticulous management of perioperative complications, and adherence to long-term follow-up are critical in enhancing the prognosis.

To our knowledge, this is the first report to establish the association between the RNF213 genotype and long-term unfavorable outcomes after EDAS in Chinese pediatric patients with MMD. Given the unclear origin of MMD and the significant impact of RNF213 p.R4810K variant on the clinical characteristics of MMD, understanding the relationship between this genotype and long-term unfavorable clinical phenotype is crucial for the future management of this condition. However, there is a scarcity of reports on the association between the p.R4810K founder variant of RNF213 and long-term unfavorable clinical outcomes after EDAS in Chinese pediatric patients. Although some studies have suggested that the p.R4810K founder variant could potentially serve as a prognostic biomarker for MMD, there is a lack of consensus regarding the exact identification of long-term unfavorable outcomes in these reports [2, 9]. Until now, it has remained unknown whether the heterozygous R4810K genotype influences the long-term unfavorable outcomes after EDAS in pediatric patients with MMD.

An increasing number of studies have provided evidence indicating that the age of onset is a crucial factor impacting long-term adverse outcomes, suggesting that a younger age of onset corresponds to a poorer prognosis [16, 17]. Our research further strengthens and supports this conclusion. Our study also revealed that perioperative complications and stroke events during follow-up play a critical role in determining the long-term prognosis. Additionally, our findings illuminated that cerebral infarction is the most common type among perioperative complications and stroke events during follow-up. However, according to our findings, the median time for stroke events observed during the follow-up period after the initial EDAS was 4.95 years. Notably, Japanese studies have demonstrated that all stroke events took place ten years following the initial procedure [18]. The observed outcome may potentially be influenced by factors such as ethnic and regional distribution. The findings underscore the significance of lifelong medical follow-up for Chinese patients with childhood-onset MMD. The purpose of this lifelong follow-up is to enable timely surgery before reinfarction occurs, ultimately enhancing the overall prognosis of these patients.

The occurrence of stroke during preoperative, perioperative, and postoperative follow-up has a significant impact on the long-term prognosis of pediatric patients with MMD [19–21]. Vigilant monitoring and efficient management of these stroke events are crucial for optimizing long-term outcomes for patients. In this study, we classified cerebral infarction into two stages: initial onset infarction and waiting infarction.

According to the results obtained from multivariate logistic regression analysis, infarction that occurs during the waiting surgery period is identified as an independent risk factor for adverse long-term outcomes after EDAS in childhood MMD. Based on our study findings, it was observed that TIA emerged as the most frequent initial symptom. Conversely, among the cases presenting with preoperative infarction, waiting infarction was observed to be more common. Even though the occurrence of hemorrhagic MMD in children is relatively scarce, comprising only 4.63% of our cohort, it still plays a critical role in influencing long-term unfavorable outcomes, which is consistent with the results of a study conducted in Korea [22].

Surgical timing can also have an impact on the long-term adverse prognosis of MMD.

Delaying surgical intervention in MMD can lead to a higher risk of recurrent strokes and other long-term complications. For patients younger than 4 years old with a high MRA score (>5) and cerebral infarction, Hayashi et al. [21] strongly recommend surgical intervention within 2 months of diagnosis. In our study, we divided the patients into two groups based on surgical timing within or beyond 2 months. Although we observed a trend suggesting a protective effect of early surgery, the results did not reach statistical significance. This finding is consistent with our observations in clinical practice. During the acute phase of ischemic or hemorrhagic stroke, a widely recognized principle highlights the need for caution when considering early surgery due to the potential for unfavorable outcomes. This is because the brain undergoes significant changes in the immediate aftermath of a stroke, and performing surgery too soon may increase the risk of complications and hamper the natural healing process. Therefore, when determining the timing of surgery for MMD, it is crucial to take a comprehensive and individualized assessment. Various factors need to be considered, including preoperative neurological function, cerebral perfusion status, and the Suzuki stage, which evaluates the severity of the disease. Of course, variability in surgical timing and outcomes observed in our study may have been influenced by several factors, including delayed diagnosis and significant neurological deterioration in some of the patients referred to our tertiary referral center.

The RNF213 p.R4810K variant has been identified not only in MMD cases worldwide but also in association with other cardiovascular diseases and vascular risk factors, including ischemic stroke and hypertension [23]. Our previous research focused on investigating how the RNF213 p.R4810K variant relates to the clinical characteristics of MMD. The results showed that this variant is significantly associated with a higher risk of developing MMD, particularly in the Chinese pediatric population. Furthermore, individuals with this variant tend to experience an earlier onset of the disease and exhibit more severe involvement of the posterior cerebral artery (PCA) in MMD cases [24]. RNF213 p.R4810K is widely recognized as a significant risk factor for MMD and it also significantly contributes to the clinical manifestations of the disease. In this study, the results indicate a significant correlation between the R4810K genotype and some clinical phenotypes (age at onset) with poor prognosis, which in turn could indirectly affect the long-term adverse outcomes in Chinese pediatric patients, which aligns with findings from the Japanese study [25].

Our analysis focused on investigating the potential correlation between the clinical phenotype of long-term unfavorable outcomes and the RNF213 p.R4810K genotype. This exploration is aimed at determining if there is an indirect influence of the genotype on the clinical phenotype of long-term unfavorable outcomes. Our research findings indicate a strong correlation between younger age at onset and an increased probability of the initial symptom being an infarction. In addition, our research results indicate that younger onset age has a significant impact on long-term prognosis, which is consistent with previous reports in the literature [22]. These studies indicated that the R4810K genotype influenced the onset age, which in turn could indirectly affect the long-term adverse outcomes in Chinese pediatric patients.

There are still certain limitations in this study. Firstly, the retrospective study and the inclusion of only Chinese pediatric patients restrict the generalizability of the findings to other ethnicities. Moreover, the sample size was relatively small, and there were limitations in the variables considered for the multivariate analysis. Additionally, some patients referred to our tertiary referral center experienced delayed diagnosis and significant neurological deterioration, leading to variability in surgical timing, which could have influenced the study's outcomes. A notable oversight was the failure to differentiate between unilateral and bilateral EDAS, a crucial factor that may significantly influence surgical outcomes. Therefore, collecting additional long-term follow-up data is crucial to achieving a comprehensive understanding of the clinical evolution of MMD in Chinese pediatric patients who have received surgical treatment.

## 5. Conclusions

The study suggests that the p.R4810K variant is associated with the onset age of MMD in Chinese pediatric patients, potentially impacting long-term outcomes. Surprisingly low recurrent stroke rates were observed across all genotypes, including homozygous individuals for the pathogenic variant, indicating that nongenetic factors may also play a role in the course and outcomes of MMD in this population.

## Figures and Tables

**Table 1:** Summary of demographics, clinical presentation, and perioperative complication.

Baseline characteristics	No. of cases or value
Gender	
Male	130 (50.19%)
Female	129 (49.81%)
Age at onset (years)	8 (6, 12)
Onset type	
TIA	149 (57.53%)
Infarction	53 (20.46%)
Hematoma	12 (4.63%)
Headache	18 (6.95%)
Epilepsy	9 (3.47%)
Other	18 (6.95%)
Follow-up	7.73 (6.88, 8.52)
Onset → operation	7.4 (2.42, 25.28)
p.R4810K genotypes	
AA	4 (1.54%)
GA	85 (32.82%)
GG	170 (65.64%)
Hyperlipidemia	
No	236 (91.12%)
Yes	23 (8.88%)
Hyperhomocysteinemia	
No	246 (94.98%)
Yes	13 (5.02%)
Family history	
No	245 (94.59%)
Yes	14 (5.41%)
Laterality	
Unilateral	8 (3.09%)
Bilateral	251 (96.91%)
Waiting infarction	
No	172 (66.41%)
Yes	87 (33.59%)
Initial mRS	
<2	238 (91.89%)
≥2	21 (8.11%)
Final mRS	
<2	225 (86.87%)
≥2	34 (13.13%)
PCA involvement	
No	188 (72.59%)
Unilateral	33 (12.74%)
Bilateral	38 (14.67%)
Suzuki stage	
<IV	100 (38.61%)
≥IV	159 (61.39%)
Perioperative complications	
No	252 (97.3%)
Infarction	5 (1.93%)
Hematoma	1 (0.39%)
Subdural effusion	1 (0.39%)
Hematoma during follow-up	
No	258 (99.61%)
Yes	1 (0.39%)
Infarction during follow-up	
No	251 (96.91%)
Yes	8 (3.09%)

Abbreviations: TIA: transient ischemic attack; PCA: posterior cerebral artery; mRS: modified Rankin scale.

**Table 2:** Comparison of baseline characteristics and outcomes between the unfavorable and favorable outcome groups.

Variables	Favorable outcome (*n* = 222)	Unfavorable outcome (*n* = 33)	*P* value
Gender			0.833
Male	112 (50.45%)	16 (48.48%)	
Female	110 (49.55%)	17 (51.52%)	
Age at onset (years)	9 (6, 12)	6 (5, 9)	0.009
Onset with infarction			0.472
No	180 (81.08%)	25 (75.76%)	
Yes	42 (18.92%)	8 (24.24%)	
Onset with hematoma			<0.001
No	218 (98.2%)	25 (75.76%)	
Yes	4 (1.8%)	8 (24.24%)	
Onset operation	7.78 (2.6, 20.45)	9.07 (3.37, 20.93)	0.903
p.R4810K genotypes			0.692
GG	149 (67.12%)	21 (63.64%)	
GA	73 (32.88%)	12 (36.36%)	
Hyperlipidemia			1
No	202 (90.99%)	30 (90.91%)	
Yes	20 (9.01%)	3 (9.09%)	
Hyperhomocysteinemia			1
No	212 (95.5%)	31 (93.94%)	
Yes	10 (4.5%)	2 (6.06%)	
Family history			0.488
No	212 (95.5%)	30 (90.91%)	
Yes	10 (4.5%)	3 (9.09%)	
Laterality			1
Unilateral	7 (3.15%)	1 (3.03%)	
Bilateral	215 (96.85%)	32 (96.97%)	
Waiting infarction			<0.001
No	158 (71.17%)	11 (33.33%)	
Yes	64 (28.83%)	22 (66.67%)	
Initial mRS			<0.001
<2	211 (95.05%)	11 (33.33%)	
≥2	11 (4.95%)	22 (66.67%)	
PCA unilateral involvement			0.494
No	195 (87.84%)	27 (81.82%)	
Yes	27 (12.16%)	6 (18.18%)	
PCA bilateral involvement			0.151
No	193 (86.94%)	25 (75.76%)	
Yes	29 (13.06%)	8 (24.24%)	
Suzuki stage			0.327
<IV	87 (39.19%)	10 (30.3%)	
≥IV	135 (60.81%)	23 (69.7%)	
Perioperative infarction			0.007
No	221 (99.55%)	30 (90.91%)	
Yes	1 (0.45%)	3 (9.09%)	
Infarction during follow-up			0.117
No	217 (97.75%)	30 (90.91%)	
Yes	5 (2.25%)	3 (9.09%)	

Abbreviations: PCA: posterior cerebral artery; mRS: modified Rankin scale.

**Table 3:** Univariate and logistic regression analyses of risk factors for unfavorable outcome (final mRS ≥ 2).

Variables	OR [95% CI]	*P* value
Gender (ref: male)	1.08 [0.52-2.25]	0.833
Age at onset	0.87 [0.79-0.97]	0.009
Onset with infarction	1.37 [0.58-3.25]	0.474
Onset with hematoma	17.44 [4.9-62.07]	<0.001
Onset → operation (ref: <2 mons)	0.44 [0.2-0.97]	0.042
p.R4810K GA (ref: GG)	1.17 [0.54-2.5]	0.692
Hyperlipidemia	1.01 [0.28-3.61]	0.988
Hyperhomocysteinemia	1.37 [0.29-6.54]	0.695
Family history	2.12 [0.55-8.14]	0.274
Laterality (ref: unilateral)	1.04 [0.12-8.75]	0.97
Waiting infarction	4.94 [2.26-10.77]	<0.001
Initial mRS (ref: <2)	38.36 [14.93-98.61]	<0.001
Unilateral PCA involvement	1.6 [0.61-4.24]	0.34
Bilateral PCA involvement	2.13 [0.88-5.17]	0.095
Suzuki stage (ref: <4)	1.48 [0.67-3.27]	0.329
Perioperative infarction	22.1 [2.23-219.35]	0.008
Infarction during follow-up	4.34 [0.99-19.09]	0.052

Abbreviations: CI: confidence interval; OR: odds ratio; PCA: posterior cerebral artery; mRS: modified Rankin scale.

**Table 4:** Multivariate and logistic regression analyses of risk factors for unfavorable outcome (final mRS ≥ 2).

Variables	OR [95% CI]	*P* value
Age at onset	0.82 [0.7-0.96]	0.014
Onset with hematoma	12.76 [1.52-106.89]	0.019
Onset → operation	0.53 [0.17-1.71]	0.288
Waiting infarction	5.69 [1.24-26.2]	0.025
Initial mRS	24.53 [6.51-92.41]	<0.001
Bilateral PCA involvement	2.22 [0.56-8.78]	0.256
Perioperative infarction	22.16 [1.45-337.96]	0.026
Infarction during follow-up	14.5 [2.04-103.12]	0.008

Abbreviations: CI: confidence interval; OR: odds ratio; PCA: posterior cerebral artery; mRS: modified Rankin scale.

**Table 5:** Summary of longitudinal surgical outcome.

Variables	No. of cases or percentages
Follow-up period	7.73 (6.88, 8.52) years
Newly developed stroke event (≥30 days after operation)	9
Infarction	8
Silent infarction	2
Symptomatic infarction	6
Median time of infarction during follow-up	4.95 (3.96, 7.19) years
Hemorrhage	1
Annual symptomatic stroke rate in the operated hemisphere	0.35%
Infarction	0.30%
Hemorrhage	0.05%

## Data Availability

Original data were generated and stored at the Fifth Medical Center of the People's Liberation Army of China (PLA) General Hospital. Data supporting these results may be obtained from the corresponding authors if the requirements are reasonable.
